# Assessment of the Feasibility, Acceptability, and Impact of Implementing Seasonal Malaria Chemoprevention in Nampula Province, Mozambique: Protocol for a Hybrid Effectiveness-Implementation Study

**DOI:** 10.2196/27855

**Published:** 2021-09-15

**Authors:** Alexandra Wharton-Smith, Kevin Baker, Arantxa Roca-Feltrer, Maria Rodrigues, Sol Richardson, Craig A Bonnington, Christian Rassi, Madeleine Marasciulo, Sonia Enosse, Francisco Saute, Pedro Aide, Eusebio Macete, Baltazar Candrinho

**Affiliations:** 1 Malaria Consortium London United Kingdom; 2 Department of Global Public Health, Karolinska Institute Stockholm Sweden; 3 Malaria Consortium Maputo Mozambique; 4 National Institute of Health (Instituto Nacional de Saúde) Maputo Mozambique; 5 Centro de Investigação em Saúde de Manhiça Manhiça Mozambique; 6 National Directorate of Public Health, Ministry of Health Maputo Mozambique; 7 The National Malaria Control Program, Ministry of Health Maputo Mozambique

**Keywords:** malaria, seasonal malaria chemoprevention, sulfadoxine-pyrimethamine amodiaquine, resistance, children under five, implementation research, Mozambique, Africa, mobile phone

## Abstract

**Background:**

Malaria is a significant cause of morbidity and mortality in children aged under 5 years in Mozambique. The World Health Organization recommends seasonal malaria chemoprevention (SMC), the administration of four monthly courses of sulfadoxine-pyrimethamine (SP) and amodiaquine (AQ), to children aged 3-59 months during rainy season. However, as resistance to SP is widespread in East and Southern Africa, SMC has so far only been implemented across the Sahel in West Africa.

**Objective:**

This protocol describes the first phase of a pilot project that aims to assess the protective effect of SP and AQ when used for SMC and investigate the levels of molecular markers of resistance of *Plasmodium falciparum* to antimalarial medicines in the study districts. In addition, it is important to understand whether SMC is a feasible and acceptable intervention in the context of Nampula Province, Mozambique.

**Methods:**

This study will adopt a hybrid effectiveness-implementation design to conduct a mixed methods evaluation with six objectives: a molecular marker study, a nonrandomized controlled trial, an analysis of reported malaria morbidity indicators, a documentation exercise of the contextual SMC adaptation, an acceptability and feasibility assessment, and a coverage and quality assessment.

**Results:**

Ethical approval for this study was granted by the Mozambican Ministry of Health National Bioethics Committee on September 15, 2020. Data collection began in October 2020, and data analysis is expected to be completed by August 2021.

**Conclusions:**

This research will make a unique contribution to our understanding of whether the combination of SP and AQ, when used for SMC, can confer a protective effect against malaria in children aged 3-59 months in a region where malaria transmission is seasonal and SP resistance is expected to be high. If the project is successful, subsequent phases are expected to provide a more comprehensive assessment of the effectiveness and sustainability of SMCs.

**International Registered Report Identifier (IRRID):**

DERR1-10.2196/27855

## Introduction

### Background

An estimated 409,000 people die from malaria each year worldwide, and children aged under 5 years are particularly vulnerable, comprising 67% of all malaria deaths in 2019 [1]. The *high burden to high impact* approach, supported by the World Health Organization (WHO), the Roll Back Malaria Partnership to End Malaria and other partners, aims to prevent disease and save lives through strategies targeted to the contextual needs of 11 countries that together account for more than 70% of the world’s malaria burden [2]. Mozambique has one of the highest incidence rates and absolute annual number of malaria cases globally [3]. Malaria causes 29% of all deaths and 42% of deaths among children aged under 5 years in Mozambique, rendering it the most significant national public health threat [4]. Mozambique has adopted the *high burden to high impact approach*, and the National Malaria Control Programme is working with partners toward the global vision of a malaria-free world. The National Malaria Control Program’s strategic plan for 2017-2022 focuses on burden reduction in highly endemic areas and on sustaining gains in low transmission areas toward elimination [5].

### Implementation of Seasonal Malaria Chemoprevention in Africa

Seasonal malaria chemoprevention (SMC) is “the intermittent administration of full treatment courses of an antimalarial medicine during the malaria season to prevent malarial illness, with the objective of maintaining therapeutic antimalarial drug concentrations in the blood throughout the period of greatest malarial risk” [6]. The currently recommended antimalarials for SMC are sulfadoxine-pyrimethamine (SP) and amodiaquine (AQ), which is administered monthly to children aged 3-59 months during the peak malaria transmission season, which typically coincides with the rainy season. A full course of SP and AQ (SPAQ) consists of 1 dose of SP and 3 doses of AQ, which are administered over a 3-day period. SMC typically involves four monthly cycles of SPAQ administration over the course of the malaria transmission season, which is referred to as a full *round*. The intervention can reduce the incidence of clinical episodes and severe malaria by approximately 75% [7]. It has also been shown that the intervention can be delivered safely at scale, achieving high coverage. The Achieving Catalytic Expansion of SMC in the Sahel (ACCESS-SMC) project scaled up SMC in 7 countries between 2015 and 2017, with few adverse drug reactions reported. SMC was associated with a protective effectiveness of 88% over 28 days in case-control studies conducted as part of ACCESS-SMC [8]. In Burkina Faso and The Gambia, the implementation of SMC was associated with reductions in the number of malaria deaths in hospitals during the high-transmission period, of 42% and 57%, respectively [8]. SMC could also avert millions of cases and thousands of deaths among children living in areas with highly seasonal malaria transmission [3]. In terms of cost, a multicountry cost-effectiveness analysis found that the weighted average economic cost of administering four monthly SMC cycles was US $3.63 per child, and ultimately, that SMC is a highly cost-effective intervention that substantially reduces malaria diagnostic and treatment costs [9].

SMC has been recommended by the WHO since 2012, for use in areas where more than 60% of annual malaria incidence occurs within 4 consecutive months, where there is a high burden of malaria in children, and where SPAQ retain their antimalarial efficacy [6,10]. To date, SMC has mainly been implemented in the Sahel region of sub-Saharan Africa, where *Plasmodium* falciparum is sensitive to both antimalarial medicines used in SMC. In 2019, 21.7 million children were targeted in 13 countries [1].

However, there is a potential risk of enhancing drug resistance and the potential for impaired development of naturally acquired immunity in children [11]. The impact of SMC on the immune response to malaria, possibly increasing the burden of malaria in later life, has not yet been proven. Studies have found that administering SMC in early life does not negatively affect the development of naturally acquired antibody responses to malaria [12,13]. In addition, there may be broader benefits of SMC, with lower parasitemia reported in health districts receiving SMC [14,15].

Challenges include the logistical burden of SMC distribution, particularly during the rainy season when access to remote areas may be compromised; however, adopting a decentralized, integrated approach through community-based distributors may support the sustainability of SMC [14]. The potential risk of development of drug resistance should be investigated further, which may pose a risk of suboptimal adherence [14]; however, the multicountry observational ACCESS-SMC study found that molecular markers of resistance occurred at very low levels [8]. Nonetheless, a few studies have found adherence to be an issue; for example, Ding et al [16] reported complete adherence in less than 20% of children receiving SMC in Niger for a full 3-day course in each cycle for four cycles.

### Drug Resistance and SMC Efficacy

The WHO recommends that SMC is suitable in areas where the efficacy of SPAQ combination remains over 90% [6]. Resistance to SP or AQ may reduce the efficacy of SMC in protecting children against clinical malaria, although the relationship between the degree of resistance and the effectiveness of SMC has not yet been clearly defined. SP efficacy is threatened by drug resistance due to mutations in the dihydrofolate reductase (*dhfr*) and dihydropteroate synthetase (*dhps*) genes [17]. According to a study conducted in Mozambique, the prevalence of *dhfr* and *dhps* mutations was 5%-6% [18], with more recent research suggesting this may be as high as >80% [19]. However, there is a paucity of data on the prevalence of SP resistance across Mozambique.

Clinical responses to SP are seriously compromised in many regions of the world, and SP is no longer recommended for the treatment of malaria episodes. However, it has been difficult to determine whether the efficacy of SP for chemoprevention is also compromised [20]. Extant evidence from intermittent preventive treatment in pregnancy suggests that the presence of resistance to SP may undermine therapeutic effectiveness [21-23]. However, SPAQ will likely still provide benefit, even when there is a high prevalence of resistance [24], and a systematic review reported that intermittent preventive treatment in pregnancy with SP protection against low–birth-weight outcomes is sustained even in areas with high levels of the quintuple mutant [25].

The Ministry of Health, National Malaria Control Programme Midterm Review of the Malaria Strategic Plan 2017-2022 has recommended SMC as a malaria control strategy to decrease transmission and accelerate impact in the highest burden locations [26]. This is in line with the WHO recommendation for individual approaches to implementing SMC based on local contexts and integrating delivery to existing programs and networks as much as possible, maximizing the potential use of community health workers and community volunteers [27]. Using community-based distributors for SMC also increased community members’ trust in the intervention [28]. Strong health communication is required to ensure that community members understand and accept that SMC is a preventive intervention, especially as qualitative research in Ghana found that people could interpret the mass distribution of medicines for the purpose of curing symptoms rather than for prevention [28].

### Study Rationale

Given the potential impact of SMC to avert many malaria infections and deaths, it is essential to investigate the role of drug resistance on the protective effect of SMC in Mozambique and to assess the feasibility and acceptability of implementing SMC in this context. In collaboration with the National Malaria Control Programme in Mozambique, Malaria Consortium will pilot SMC from November 2020 to February 2021, to a target population of approximately 72,000 children aged under 5 years in 2 districts of Nampula Province.

### Study Aims

We will evaluate the SMC pilot with two primary aims: to determine the protective effect of SPAQ when used for SMC in this context and to assess the feasibility and acceptability of implementing SMC in terms of coverage, quality, and stakeholder perceptions. The study objectives are as follows: (1) to determine the baseline prevalence of SPAQ resistance and any increase in resistance prevalence after one annual round of SMC, (2) to determine whether receipt of SPAQ is associated with a reduction in the odds of clinically significant malaria outcomes, (3) to assess the change in reported malaria morbidity indicators through routine data, (4) to document the adaptation of SMC implementation to the Mozambican context, (5) to explore the feasibility and acceptability of SMC among stakeholders, and (6) to evaluate the process of SMC implementation in terms of distribution quality and coverage.

## Methods

### Study Setting

**Figure 1:**
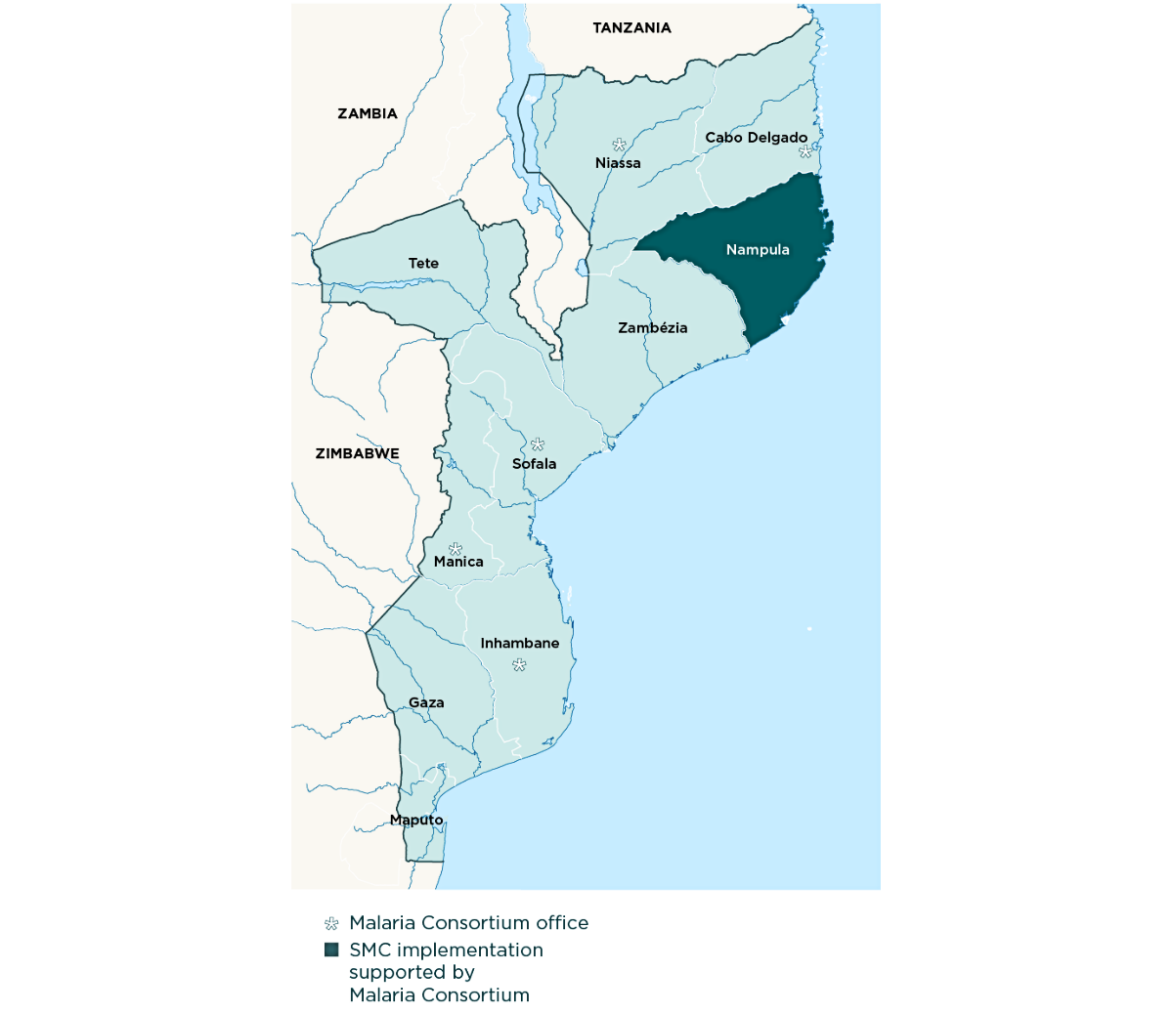
Nampula Province, Mozambique, where the study will be conducted. SMC: seasonal malaria chemoprevention.

**Figure 2:**
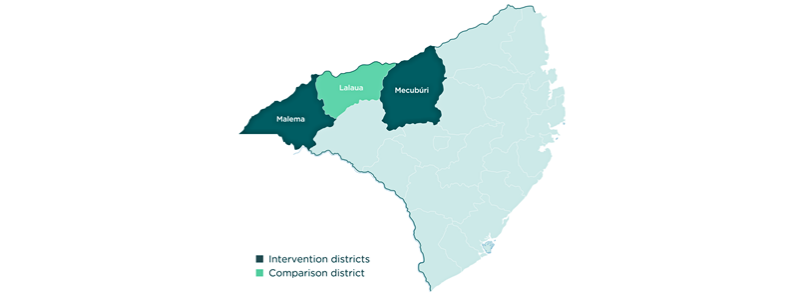
Seasonal malaria chemoprevention intervention and comparison districts.

The study will be conducted in Malema, Mecuburi, and Lalaua districts in Nampula Province, northeastern Mozambique (Figures 1 and 2). Several key informant interviews may be conducted with stakeholders based in Maputo.

To identify suitable districts for SMC, an SMC suitability ranking was conducted by the WHO for all provinces. These criteria included a variety of factors such as (1) seasonality eligible for SMC (60% of rainfall concentrated in 4 months), (2) mortality (areas of highest under-five mortality using health management information system) data, (3) access to care (highest ranking given to areas where access to care was poor), and (4) treatment-seeking behavior (highest ranking given to areas where treatment-seeking behavior was poor). Using these four main categories, the average will be calculated to estimate the final ranking and identify the top 20 suitable districts to maximize the impact of SMC. From the list of suitable districts, an additional consideration was taken given the importance of implementing the intervention in an area where no other new interventions were taking place so that an evaluation could be implemented aiming at attributing change to the intervention under investigation. Hence, Malema and Mecuburi districts in Nampula Province were selected, with Lalaua as a comparator, as no indoor residual spraying or new long-lasting insecticidal nets were targeted in these areas, ensuring a robust evaluation component for this pilot study.

### Study Populations

The study population that is eligible to receive SMC includes afebrile children of either sex, aged 3-59 months, for any of the SMC cycles, residing in Malema and Mecuburi districts. In addition, for the purpose of the end-of-round survey, which includes an indicator measuring the age eligibility of enrolled children, we included children residing in the aforementioned districts, aged 5-10 years, who may inadvertently have received SMC. In addition, health workers who are involved in SMC implementation, caregivers of children aged under 10 years, community leaders, and key stakeholders such as health officials at different levels of the health system and those involved in SMC implementation will be sampled, according to the study objective, described in more detail in the *Methods* section. The Lalaua district will serve as a control area (see Table 1 for estimated population sizes).

**Table 1:** Estimated target population of seasonal malaria chemoprevention implementation districts and comparison district by age (2020).

Target population estimates		Population of children aged 3-11 months, n (%)	Population of children aged 12-59 months, n (%)	Total population, n	
**Intervention area**					
	Malema	4031 (11.04)	32,438 (88.94)	36,469	
	Mecuburi	3959 (11.16)	31,515 (88.83)	35,474	
Total SMC^a^ target population					71,943
**Control**					
	Lalaua	1978 (10.74)	16,425 (89.25)	18,403	

^a^SMC: seasonal malaria chemoprevention.

### Study Design

We will adopt a type 2 hybrid effectiveness-implementation study design that evaluates the effects of a clinical intervention on relevant outcomes while collecting information on implementation [29]. The hybrid effectiveness-implementation study design supports the pursuit of different lines of research simultaneously, which facilitates the more rapid translation and uptake of study findings for policy makers and implementers [29,30]. This study will use mixed methods to address the aforementioned objectives. Assessments of the protective effect of SPAQ, as delivered as part of SMC implementation, will provide evidence on whether this is appropriate in a region where SP resistance is suspected. Evaluating the implementation of SMC will generate knowledge on the feasibility and acceptability of this intervention in situ, including the challenges, barriers, and facilitators in the local context. Such findings can be useful to inform which intervention components are generalizable and which require local adaptation for other settings [31].

**Figure 3:**
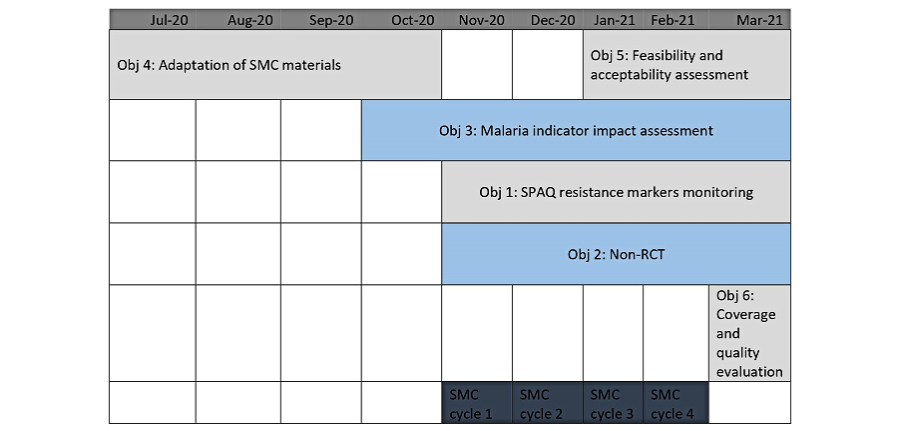
Timeline of seasonal malaria chemoprevention study objectives. Obj: Objective; RCT: randomized controlled trial; SMC: seasonal malaria chemoprevention; SPAQ: sulfadoxine-pyrimethamine and amodiaquine.

Four monthly cycles of SMC drugs will be distributed door-to-door to eligible children aged between 3 and 59 months by community distributors between November 2020 and February 2021 in 2 districts of Nampula Province. SMC tools and protocols used in Sahelian countries where the Malaria Consortium supports SMC delivery will be adapted to the context in Mozambique for this purpose. A third district will serve as a comparison district (the latter will receive standard malaria prevention, control, and case management). Data collection across the study components was conducted between October 2020 and May 2021 (Figure 3).

The methods are organized according to the study objective, described in more detail below, with an overview provided in Table 2 and Figure 3.

**Table 2:** Overview of research objectives, methods, and sampling.

Objective	Method	Study population	Inclusion criteria	Exclusion criteria	Sample size (N=3320), n (%)
SPAQ^a^ resistance molecular marker monitoring	Quantitative: molecular analysis	Eligible children aged 3-59 months on the day 1 of the first SMC^b^ cycle	Aged 3-59 months attending the health facilities in study area with fever or history of fever in preceding 24 hours, with a positive malaria RDT^c^ and, with prior informed consent obtained from the parents or guardians	Presence of any signs or symptoms of severe malaria, refusal to participate	600 (18.07)
Nonrandomized controlled trial	Quantitative: nonrandomized controlled trial	Eligible children aged 3-59 months on the day 1 of the first SMC cycle	Eligible children, visited home for enrollment	Presence of a severe, chronic illness and a history of a significant adverse reaction to sulfadoxine-pyrimethamine or amodiaquine Refusal to participate	800 (24.09)
Assessment of impact of SMC on reported malaria morbidity indicators	Quantitative: DHIS2^d^ or SISMA^e^ data analysis	Data quality audit and morbidity analysis of all confirmed malaria cases reported in children aged less than 5 years in the 3 study districts from November 2020 to March or May 2021	HMIS^f^ data on health facility attendance for children aged 0-13 years (inclusive) will be extracted from health facility registers as full line listings, covering November 2020 to May 2021	Data from children aged >13 years or those falling outside the specified dates	26 health facilities in all 3 study districts
Process documentation of SMC adaptation	Qualitative: documentation of the adaptation process	N/A^g^	N/A	N/A	N/A
Feasibility and acceptability assessment	Qualitative: KIIs^h^ and FGDs^i^	Health workers implementing SMC Caregivers of children aged 3-59 months, community members, and community leaders Health workers SMC implementers Health officials and policy makers (district, provincial, and national)	Must fall into one of the study population’ categories, must consent to participate	Not categorized into one of the described study populations Refusal to participate	120 (3.61)
Coverage and quality evaluation	Quantitative: end-of-round survey	Eligible children aged 3-119 months who have been a resident in the study location for a minimum of at least one month, during the SMC pilot implementation period. Survey responses will be provided by caregivers	Households with children aged 3-119 months, resident in the study location >1 month during SMC Aged 18 years or older with the primary responsibility of feeding and daily care of at least one child aged 3 months to 10 years	No one aged 18 years or older available at the time of data collection No children aged 3-119 months present Refusal to participate	1800 (54.22)

^a^SPAQ: sulfadoxine-pyrimethamine and amodiaquine.

^b^SMC: seasonal malaria chemoprevention.

^c^RDT: rapid diagnostic test.

^d^DHIS2: district health information software.

^e^SISMA: Sistema de informação em Saúde e Monitoria e Avaliação.

^f^HMIS: health management information system.

^g^N/A: not applicable.

^h^KII: key informative interview.

^i^FGD: focus group discussion.

### Ethical Considerations

Ethical approval for this study was granted by the Mozambican Ministry of Health National Bioethics Committee on September 15, 2020. Only participants who met the inclusion criteria and provided written informed consent will be included.

### Study Objectives and Methods: SPAQ Resistance Molecular Marker Monitoring

#### Overview

The purpose of this study is to determine the baseline prevalence of SPAQ resistance markers and any eventual increase after one round (four monthly cycles) of SMC as assessed via molecular markers in the population. Specifically, to detect prevalence over time in the proportion of symptomatic children, using a positive rapid diagnostic test (RDT), residing in 2 districts where SMC will be implemented who carry parasites with *Plasmodium falciparum* dihydrofolate reductase *(dhfr), Plasmodium falciparum* dihydropteroate synthase (*dhps*)*, Plasmodium falciparum* chloroquine resistance transporter gene (*pfcrt*) and/or *Plasmodium falciparum* multidrug resistance gene 1 (*pfmdr1*) mutations, compared with children living in a neighboring district with similar epidemiologic characteristics but not receiving SMC. Children enrolled in both areas will be aged 3-59 months and will have similar clinical and parasitological characteristics. Health facility–based cross-sectional surveys will be conducted in October 2020, before the SMC distribution begins (baseline), and in March 2021, after one complete round of SMC (endline), to measure the prevalence of molecular markers associated with resistance to SPAQ in symptomatic children aged less than 5 years with a positive RDT attending selected health facilities in the intervention and control areas. Monitoring the prevalence of alleles associated with drug resistance will be done by collecting blood samples from symptomatic children with evidence of infection. During the surveys, fingerpick blood samples will be collected on Whatman 903 (10534612) filter papers (dried blood spots). Sample collection will be performed in 2 selected first-level health facilities in each district of the intervention area and also in 4 selected health facilities in the comparator district, with a total of 8 health facilities across all 3 districts. The main outcome measure is the prevalence of molecular markers associated with SP (codons 108, 51, and 59 in *dhfr* and 437, 540, and 581 in *dhps*) and AQ (codons 72-76 in *pfcrt* and 86, 184, and 1246 in *pfmdr1*) resistance in blood samples collected from symptomatic children aged less than 5 years with a positive RDT attending the selected health facilities. The prevalence will be assessed in areas with SMC and with no SMC at baseline and at the end of the project.

#### Indicative Sample Size Calculations

**Figure 4:**
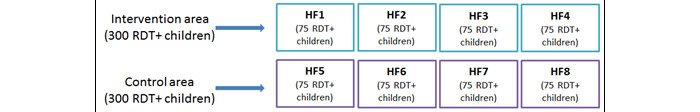
Estimated sample sizes. Numbers correspond to the children with positive malaria test results. HF: health facility; RDT: rapid diagnostic test.

The sample size will be determined using the WHO protocol for drug efficacy testing [32]. The survey’s sample size will be calculated to estimate changes in the prevalence of SPAQ resistance markers with sufficient precision to detect a statistically significant difference before and after SMC implementation. It is assumed that the prevalence of *dhfr and dhps* sextuple mutants is 0% [33] in the intervention area. A sample size of 242 samples per survey per arm was estimated to have 90% power to detect a difference at the 5% level (*P*=.05) between baseline and endline. This will permit confirmation of a prevalence of 5% or higher in *dhfr and dhps* sextuple mutants. Assuming a 10% loss of samples or uninterpretable analysis, the number will be rounded up to 300 samples per area (intervention and comparator districts) per survey per country. Figure 4 shows the estimated sample sizes for each health facility. Samples will be collected in 8 health facilities (4 in the intervention area and 4 in the comparator *control* area). 

#### Selection of Survey Health Facilities

Samples will be collected from selected health facilities from the list of all health facilities in the control and intervention areas, based on attendance of children with fever or history of fever and prevalence of *Plasmodium falciparum* infection among them based on routine data reporting.

#### Study Population

The study population will include children aged 3-59 months attending the selected health facilities in both the intervention and comparator districts. The number of children to be screened in each health facility will depend on the proportion of malaria RDT-positive cases over the total number of children meeting the eligibility criteria. Children who do not meet the inclusion criteria, for example, those with a negative RDT result, will be referred within the same health facility for further assessment and appropriate treatment.

#### Sample Collection and Molecular Analysis

Caregivers of children meeting all the inclusion criteria mentioned earlier will be interviewed using a short paper questionnaire to record personal identifiers of the children such as date of birth, sex, date of interview, and residence location. The study staff will approach them as part of their clinical visits.

A blood sample (4 drops) will be collected through finger prick onto a filter paper (Whatman 3MM) to determine the prevalence of *dhfr*, *dhps*, *pfcrt*, and *pfmdr1* mutations. Other molecular markers of antimalarial resistance will be considered for analysis as comparators. Specimens will be labeled anonymously (unique identifying code, study health facility, and date), dried, stored in individual plastic bags with desiccants, and protected from light, humidity, and extreme temperature until analyzed. All samples will be batched and stored at 4 °Cat the Centro de Investigação em Saúde de Manhiça laboratory (until complete sample collection and analysis). *Dhfr*, *dhps, pfcrt, and pfmdr1* genotypes will be determined using nested mutation-specific polymerase chain reaction, sequencing, and/or polymerase chain reaction-restriction fragment length polymorphism. The prevalence of *dhfr* mutations at codons 51, 59, and 108 and *dhps* mutations at codons 431, 437, 540, 581, and 613 and *pfcrt* codons 72-76 and *pfmdr1* codons 86, 184, and 1246 will be calculated among the sampled children, and differences in the proportions of samples with each of these mutations between baseline and endline will be tested using the *z* test.

### Nonrandomized Controlled Trial

#### Overview

A nonrandomized controlled trial will be conducted to determine whether the receipt of SPAQ is associated with a reduction in the odds of clinically significant malaria outcomes and to estimate the protective effect of SPAQ. There will be two arms: one (control arm) in the comparator district and the other in one of the SMC intervention districts. Communities will be randomly selected in both the intervention and control districts. Eligible children (aged 3-59 months at the first SMC cycle) will be recruited through random selection. Exclusion criteria include the presence of a severe, chronic illness and a history of a significant adverse reaction to SP or AQ. Selected children will be treated appropriately and retained during the trial. In the control district, communities bordering the intervention district will be avoided because of potential unintended *leakage* of SPAQ across the district boundary.

#### Sample Size

The study will be powered to have an 80% chance of detecting a 40% reduction in incidence with statistical significance at the 5% level. It is assumed that study participants, in the absence of SMC, would experience 0.2 clinical episodes per child per high-transmission season (corresponding to the SMC round) of sufficient severity to present to a health facility [10]. The sample size calculation, based on the formula for a binary outcome superiority trial, shows that a sample of 654 eligible children (or 327 per arm) is required to provide sufficient statistical power. This multiplies to 818 under the assumption of a 20% loss to follow-up over the four cycles. Therefore, we aim to recruit a minimum of 800 eligible children (400 per arm).

#### Primary Outcome

The primary outcome of this study will be visits to a health facility for suspected malaria by eligible children and confirmation of malaria diagnosis using an RDT during the 5-month study period, which includes a period of 28 days after the final administration of SPAQ.

#### Recruitment and Data Collection

Households will be randomly sampled from selected communities in both the control and intervention arms and 1 eligible child recruited at random in each household. A short baseline questionnaire will be administered to collect individual data on each child and to confirm their eligibility after the caregivers provided their consent. In the intervention arm, the questionnaire will be administered before the intake of SPAQ for SMC. The primary end point will be recorded through passive surveillance by study clinicians in both the intervention and comparator areas during the 4-month study period. Children recruited into the study presenting at clinics will be identified using information on SMC record cards, on which a child’s information and SMC doses are recorded, and data on health facility visits including suspected malaria cases and results of RDTs will be matched to baseline questionnaire data to build a database for analysis.

#### Data Analysis

Data will be analyzed using multivariate logistic regression with odds ratios estimated for the association between allocation to the intervention arm and (suspected and confirmed) malaria diagnosis at any point during the study period, converted to a percentage estimate of protective effect. To compensate for the lack of preintervention random allocation of participants to intervention or control groups, covariate adjustment will be made for potential confounders that may influence respondents’ odds of experiencing a clinically significant case of malaria. Covariates, measured at baseline, will include sex, age, parental education, and long-lasting insecticidal net use and all of these will be operationalized as categorical variables. Analyses will also use a multivariate Cox proportional hazards model with multiple Cox regression. *Failure* defined as a visit to a health facility for suspected malaria and/or a confirmed case of malaria and timing of this event based on the original date of health facility attendance during the study period and the estimated protective effect calculated.

### Assessment of SMC Distribution Impact on Reported Malaria Morbidity Indicators Using Routine Data

We will investigate the impact of SMC on reported malaria morbidity (as a primary end point) in children aged 3-59 months in the study area. Differences in rates of malaria incidence by age at the health facility level will be investigated using a quasi-experimental design based on regression discontinuity analysis, taking advantage of the discontinuity in eligibility for SMC based on age.

Before obtaining data on malaria indicators at the health facility and district levels, a data quality audit will be conducted to assess the quality of the data that will be used for analysis. The quality of data from districts where SMC is implemented will also be compared with that of data from the comparator district. Health management information system data on health facility attendance for children aged 0-13 years (inclusive) will be extracted from health facility registers as full line listings, covering November 2020 to May 2021 in both implementation districts and the control district by photographing the relevant pages of the registers and entering data directly into Excel tables as appropriate. While monthly suspected malaria cases presenting at each health facility will be calculated, other variables including date of health facility attendance, sex, date of birth (or closest estimate of age in months as a preference, or age in years if this cannot be confirmed from an identity document, previous health facility record, or vaccination or medical card), whether a malaria test was performed, the type of test performed (RDT or microscopy), test results (confirmed malaria or negative test results), malaria mortality, and any other variables collected routinely for all child attendees (eg, nutrition status) will be obtained. Observations from registers will be categorized by children’s ages according to the smallest possible increments. Incidence curves [34] for suspected and confirmed malaria cases by age will be modeled from these data (using offset terms to adjust for estimated numbers of children in each age category by health facility catchment area). Incidence curves by age will either be fitted with quadratic or cubic regression terms in Stata 16 (StataCorp) or using a thin plate spline term in R (R Foundation for Statistical Computing; using the mixed gam computation vehicle package). Regression discontinuity analysis [35] using negative binomial models will then be used to assess the reduction in malaria incidence by age-dependent eligibility for SMC. As the primary exposure variable, eligibility will be coded as a binary variable (1=3-59 months 0=0-2 months and 60-155 months) based on age at the start of the SMC round and district. Adjustments will be made for the month of health facility attendance. If possible, random effects will be fitted, with observations of monthly malaria cases nested within clinics. The results will be expressed as rate ratios for the difference in incidence in the eligible age group compared with the expected incidence curve. Models will adjust for sex as an interaction term (considered a moderator for age-dependent malaria susceptibility). Sensitivity analyses may consider the effects of unintended coverage of children aged 5-9 years.

### Process Documentation of SMC Adaptation

The purpose of this component is to systematically capture the process of how the SMC model implemented by the Malaria Consortium and partners in West and Central African countries is adapted to the Mozambican context. Specifically, this will take into consideration the existing government health system, structures, personnel, and service delivery at different levels. In view of the current COVID-19 pandemic, all of the aforementioned activities and tools will be appropriately adapted to this outbreak context, which will constitute an additional aspect to be documented as part of the model adaptation. Previous study protocols, implementation reports, tools, and materials from other countries where the Malaria Consortium has implemented SMC will be reviewed and collated. Relevant materials will be adapted and translated for the Mozambican context. All of these adaptations will be captured using a template process documentation tool.

### Feasibility and Acceptability Assessment

A key component of investigating the feasibility and efficacy of SMC in Mozambique is understanding the views and experiences of those involved in its implementation and use.

To explore the feasibility and acceptability of implementing SMC, a qualitative assessment using key informant interviews and focus group discussions (FGDs) will be conducted. This study component will examine how SMC is viewed, experienced, and engaged with by different stakeholder groups, including policy makers, implementers, and beneficiaries.

The study population includes health workers and community volunteers who are involved in SMC implementation, caregivers of children aged 3-59 months, community members, community leaders, and key decision makers such as health officials at different levels of the health system. Purposive approaches to sampling will be adopted to include those best able to provide insight into the topic being explored and a range of views and experiences relating to SMC [36]. Participants will fall under four main groups: caregivers of children eligible for SMC; health workers and community volunteers involved in SMC delivery; community members in areas where SMC is implemented; and key informants involved in SMC implementation, program management, and policy making.

Within each participant group, participants with a range of different experiences will be identified, where possible. For example, for caregivers, efforts will be made to include participants of different genders, ages, living in more rural and urban locations; those considered more marginalized, such as adolescents, older caregivers, and people living remotely; and those with different socioeconomic status and education levels. Although we presume that most caregivers will be female, we will attempt to include male caregivers where possible and relevant. We will also aim to include those with different experiences relating to SMC, that is, those who refused and those who agreed to receive SPAQ for their children. For health workers and community-based volunteers, those of different ages and experience and seniority levels will be identified for recruitment. For community members, those with potentially different perspectives will be included, for example, village leaders, older men, older women, and younger women. For key informants, implementing partners and those from district, provincial, and national levels and with a range of different potential insights, for instance, relating to malaria prevention policy making, program management, and drug supply chain management, will be identified for recruitment. In addition to identifying participants purposively, the end-of-round approaches to sampling will be adopted, with the final sample size determined based on evidence of achieving data saturation, when additional participants do not generate new findings relating to the topic of investigation [37,38]. We estimate recruiting approximately 10-20 participants per group (Table 3) for participation in an in-depth interview. In addition, 6-12 FGDs will be conducted with approximately 6-8 participants per group.

Data will be generated through in-depth interviews and FGDs, based on topic guides, to explore views on SMC, areas relating to SMC acceptability, and reflections on the implementation experience. Topic guides will serve as prompts for the interviewer but will be flexible and participant led. In-depth interviews will aim to encourage participants to elaborate and provide in-depth accounts of their views and experiences relating to SMC, or *thick descriptions* [39], including through using nonverbal and verbal probing. Topic guides will vary the areas of focus, depending on the participant group. For example, interviews with caregivers will explore topics including general views on malaria, malaria prevention options, and SMC, as well as perceived malaria risk, perceived benefits and costs or negatives of SMC, concerns, challenges, and support. Interviews with community distributors will explore topics such as experiences administering SPAQ, how community members respond to SMC, what community distributors themselves think about the pros and cons of SMC and its appropriateness in the region, challenges, and support for engagement with it. Interviews with key informants will explore topics relating to implementation views and experiences, including reflections on the pilot implementation, the appropriateness of SMC for preventing malaria in the region and country, and challenges and opportunities for national rollout.

**Table 3:** Sampling frame.

Participant group and estimated sample size		Data collection technique
**Caregivers**		
	10-20	IDI^a^
	3-6	FGD^b^
**Health workers and community-based volunteers**		
	10-12	IDI
	3-6	FGD
**Key informants or stakeholders**		
	10-12	IDI
**Community members**		
	3-6	FGD

^a^IDI: in-depth interview.

^b^FGD: focus group discussion.

FGDs can facilitate an understanding of social norms, providing access to a range of perspectives [40], and the interaction between participants [41]. FGDs will therefore enable the exploration of wider views on SMC acceptability in the communities where the pilot is implemented, as well as views regarding different malaria prevention options, decision-making processes relevant to SMC, and potential barriers and facilitators for SMC implementation. FGDs with community members and caregivers will be homogenized by gender and age to facilitate interaction among participants and the expression of norms and consensus among peers in a grouping that is sensitive to cultural norms. All interviews and FGDs will be audio recorded and then transcribed and translated verbatim. Data collection and analysis will be conducted iteratively, with data analysis beginning at the point of data generation and with participant recruitment and topic focus being adapted as data collection progresses to further test emerging concepts and potential discrepancies from majority themes [42]. Data will be analyzed thematically using coding to identify emergent patterns, concepts, and categories from participants’ accounts.

### Coverage and Quality Evaluation (End-of-Round Survey)

#### Overview

The aim of this component is to evaluate the process of SMC implementation in terms of quality, coverage, and adherence to COVID-19 safety guidelines*.* To evaluate the coverage provided in the SMC pilot, an end-of-round survey will be conducted in March 2021. The objective of the end-of-round survey is to retrospectively determine coverage by surveying caregivers of eligible children aged 3-59 months and ineligible children aged 60-119 months, as leakage into the older age group has been anecdotally observed in other SMC countries [43]. Caregivers will be asked if their children receive the full 3-day course of the SPAQ during each cycle of the SMC round. The key indicators that will be assessed will include (1) the proportion of households with eligible children visited by a community distributor, (2) the proportion of day one SPAQ administered by community distributors to eligible children (in terms of children who received day one SPAQ at least once during 2020-2021 and by monthly cycle), (3) the proportion of eligible children who received a full 3-day course of SPAQ (including day 2 and day 3 AQ, among eligible children who received day one SPAQ), (4) the proportion of SPAQ administered by community distributors by directly observed treatment (among eligible children who received day one SPAQ), and (5) the proportion of day one SPAQ received per eligible child over the course of the SMC round (including the proportion of children who received day one SPAQ during all four SMC cycles). To measure the quality of SMC coverage, the three key indicators assessed will be the correct age eligibility included, correct directly observed therapy observed, and correct dosage administered. These quality indicators are included along with the coverage indicators in an end-of-round survey tool.

#### End-of-Round Survey Study Area

This study aims to achieve a representative sample of children aged 3-119 months in households grouped within clusters identified from health facility catchment areas across the 2 intervention districts of the SMC pilot (ie, Malema and Mecuburi).

#### Study Design

For this objective, the design will be a cross-sectional cluster randomized survey to evaluate the coverage level and quality of SMC piloted in the Malema and Mecuburi districts. Households refusing to participate in the study will be replaced with the next eligible household until the estimated sample size is attained.

#### Sample Size and Technique

The end-of-round survey will use multistage random samples of households in areas covered by the Malaria Consortium’s SMC pilot and will intend to achieve a representative sample of the target population at the district level to estimate the coverage of SMC at the level of individual eligible children. The sampling protocol aims to achieve a self-weighted sample with sampling units selected with a probability proportional to size. Only at the last stage of sampling (ie, at the household level) will a constant number of eligible children (1 child per household) be selected. The survey will be powered to provide an estimate of SMC coverage for children aged 3-59 months with a margin of error of 5%, while also providing a representative sample of children aged 60-119 months. The main sampling frame for the selection process will be a list of villages. Villages will then be randomly selected using the probability proportional to size. Villages will be the primary unit of sampling through which households and eligible children will be selected randomly. This may be reviewed once the study starts if this approach is not feasible in practice. A primary caregiver in this survey refers to any individual, aged at least 18 years, with the primary responsibility of feeding and daily care of at least one child aged less than 5 years, in a household where he or she has been a resident before the start of the SMC pilot or 1 month before the last cycle of SMC. The sample size calculation was performed using Stata 16 using the *svysampsi* command, based on the following assumptions: (1) the assumed intracluster correlation is 0.2; (2) 15 eligible children per cluster (b=cluster size); (3) the (design/cluster effect) = 1 + (b − 1) intracluster correlation = 1 + (15 − 1) 0.2 = 3.8; (4) the coverage rate of SMC in children aged 0-4 years of at least 80% (and in children aged 5-9 years of at most 20%); (5) a margin of error of 5%; (6) finite population adjustment is applied (75,000); (7) nonresponse rate of 5%; and (8) assumed ratio of children aged 5-9 years to those aged 0-4 years of 0.88.

#### Sampling Procedure

The sample size calculation revealed that a sample of 1842 children was required. It was decided that the survey would include 120 clusters, each comprising 15 children (n=1800), with 60 in each of the two districts. A constant number of households (15) will be randomly sampled from these areas, with the assumption that the populations in each sampling unit were of approximately equal size. One eligible child will be sampled from each compound in the absence of a household-level sampling frame, under the assumption that households contain a similar number of eligible children. Sampling at each stage will be conducted without replacement; once a supervision area or cluster has been selected, it should no longer be eligible for further selection.

#### Study Tools

The data were collected using a questionnaire developed by the Malaria Consortium. The survey questionnaire will be translated into Portuguese and Macua languages and uploaded into the SurveyCTO software application that will enable direct, field-based computer-aided personal interview and remote capture of the data and transfer to a netbook computer.

Although the coverage surveys will be the main method to determine coverage, two additional data sources will be analyzed and compared with survey results: (1) administrative data based on SMC tally sheets completed by distributors and data compiled via summary forms and end-of-cycle reports, and (2) (coverage=doses delivered/target population) and stock consumption data (coverage=SPAQ coblister packs received before the campaign−SPAQ coblister packs left at the end/target population).

#### Data Collection

Data will be collected by administering questionnaires to the identified respondents in the sampled compounds within the communities. The survey questionnaires will be administered by a trained research team. All surveys will be administered using SurveyCTO, an electronic data collection platform for smartphones, and data will be uploaded to a remote server after each day of data collection. Interviews will be conducted in local languages using the questionnaires provided by the Malaria Consortium, with data collectors translating from the Portuguese questionnaire on the spot and assigning responses to predefined answer categories in SurveyCTO. For the age eligibility indicator, survey respondents may be asked to present a birth certificate or vaccination card to the data collector to verify the child’s date of birth. The duration of data collection is expected to last for 7 days during the first week of March 2021. 

#### Data Analysis

Data analysis will be carried out using Stata 16. Coverage will be calculated using the proportion command. Population size weights will be applied using the svy command as appropriate for estimates of coverage indicators when it is not possible to achieve a self-weighting sample. All indicators of interest will be calculated as proportions by district and an average across both districts.

### Availability of Data and Materials

The associated study protocol and data collection tools will be made available upon request from the corresponding author. Quantitative data sets will be available from the corresponding author upon reasonable request after the completion of primary analyses and dissemination of results. Qualitative study data sets will not be available, as they may include identifiable information that could compromise participant identity.

## Results

Data collection and analysis from all six objectives will be completed by September 2021.

## Discussion

### Principal Findings

This study will provide a unique contribution to the evidence on the prevalence of SPAQ resistance in Mozambique and more broadly in the region, and to what extent it may challenge the protection that SMC confers. Furthermore, this first evaluation of SMC implementation in this context will generate insights on the enablers, challenges, perceptions, feasibility, acceptability, quality, and coverage of this intervention in situ. This study is vital, as it is the first time that SMC is implemented in East and Southern Africa.

Recent evidence from the ACCESS-SMC program showed that the protective effectiveness of each monthly treatment was similar to that observed in randomized controlled trials [44,45]. In 2 countries with district health information software-2 databases established before SMC scale-up (ie, The Gambia and Burkina Faso), estimated reductions of 57% and 42% in the number of malaria deaths in district hospitals were determined for the SMC intervention period, and reductions of 53% and 45% in the number of outpatient cases, respectively [8]. Similar reductions were observed in the number of outpatient malaria cases in other countries [8]. These results represent the first large-scale evaluation of SMC implemented by national programs and provide the first evidence of an impact on malaria deaths. Earlier studies in Burkina Faso and Mali showed effects on prevalence [46,47] and cost-saving benefits [9,48].

Molecular markers of SPAQ resistance occurred at a low prevalence in previous studies in the Sahel, consistent with the effectiveness of SMC observed in several case-control studies. However, there is evidence of selection for resistance to SP in parasites sampled from the same age group in the areas where SMC will be implemented for the first time in Mozambique. Therefore, resistance to both SPAQ needs to be monitored via standardized methods, across all regions in Mozambique where SMC is used, to provide early warning of loss of effectiveness.

### Limitations

Leakage of SPAQ to older age groups not targeted by SMC programs raises a concern for the development of drug resistance, as doses administered are unlikely to offer sufficient protection against malaria transmission. This also influences the secondary data analysis in the same way; leakage reduces the apparent effect size in the targeted age group.

### Conclusions

This research will be a novel contribution to our understanding of whether SPAQ, when used for SMC, can confer a protective effect against malaria in children aged 3-59 months in a region where malaria transmission is seasonal and SP resistance is expected to be high. In addition, the findings from this study will inform future SMC implementation in Mozambique and other countries and potentially enhance the quality of SMC distribution in terms of quality, coverage, and acceptability. Subsequent phases are expected to provide a more comprehensive assessment of the effectiveness and sustainability of SMCs.

## Abbreviations

ACCESS-SMC: Achieving Catalytic Expansion of SMC in the Sahel
AQ: amodiaquine
dhfr: dihydrofolate reductase
dhps: dihydropteroate synthetase
FGD: focus group discussion
pfcrt: Plasmodium falciparum chloroquine resistance transporter gene
pfmdr1: Plasmodium falciparum multidrug resistance gene 1
RDT: rapid diagnostic test
SMC: seasonal malaria chemoprevention
SP: sulfadoxine-pyrimethamine
SPAQ: sulfadoxine-pyrimethamine and amodiaquine
WHO: World Health Organization

## References

[1] World Malaria Report 2020. World Health Organization, 2020.

[2] High burden to high impact: a targeted malaria response.

[3] World Health Organization (2019). Global Malaria Program. World Malaria Report.

[4] Moonasar D, Maharaj Rajendra, Kunene Simon, Candrinho Baltazar, Saute Francisco, Ntshalintshali Nyasatu, Morris Natashia (2016). Towards malaria elimination in the MOSASWA (Mozambique, South Africa and Swaziland) region. Malar J.

[5] Aide P, Candrinho Baltazar, Galatas Beatriz, Munguambe Khátia, Guinovart Caterina, Luis Fabião, Mayor Alfredo, Paaijmans Krijn, Fernández-Montoya Lucía, Cirera Laia, Bassat Quique, Mocumbi Sonia, Menéndez Clara, Nhalungo Delino, Nhacolo Ariel, Rabinovich Regina, Macete Eusébio, Alonso Pedro, Saúte Francisco (2019). Setting the scene and generating evidence for malaria elimination in Southern Mozambique. Malar J.

[6] World Health Organization (2013). World Malaria Program. Seasonal malaria chemoprevention with sulfadoxine-pyrimethamine plus amodiaquine in children: a field guide.

[7] Meremikwu M, Donegan Sarah, Sinclair David, Esu Ekpereonne, Oringanje Chioma (2012). Intermittent preventive treatment for malaria in children living in areas with seasonal transmission. Cochrane Database Syst Rev.

[8] ACCESS-SMC Partnership (2020). Effectiveness of seasonal malaria chemoprevention at scale in west and central Africa: an observational study. Lancet.

[9] Gilmartin C, Nonvignon J, Cairns M, Milligan P, Bocoum F, Winskill P, Moroso D, Collins D (2021). Seasonal malaria chemoprevention in the Sahel subregion of Africa: a cost-effectiveness and cost-savings analysis. The Lancet Global Health.

[10] Cairns M, Roca-Feltrer Arantxa, Garske Tini, Wilson Anne L, Diallo Diadier, Milligan Paul J, Ghani Azra C, Greenwood Brian M (2012). Estimating the potential public health impact of seasonal malaria chemoprevention in African children. Nat Commun.

[11] Greenwood B (2017). New tools for malaria control – using them wisely. Journal of Infection.

[12] Mahamar A, Issiaka Djibrilla, Barry Amadou, Attaher Oumar, Dembele Adama B, Traore Tiangoua, Sissoko Adama, Keita Sekouba, Diarra Bacary Soumana, Narum David L, Duffy Patrick E, Dicko Alassane, Fried Michal (2017). Effect of seasonal malaria chemoprevention on the acquisition of antibodies to Plasmodium falciparum antigens in Ouelessebougou, Mali. Malar J.

[13] Quelhas D, Puyol L, Quintó L, Serra-Casas E, Nhampossa T, Macete E, Aide P, Mayor A, Mandomando I, Sanz S, Aponte Jj, Chauhan Vs, Chitnis Ce, Alonso Pl, Menéndez C, Dobaño C (2008). Impact of Intermittent Preventive Treatment with Sulfadoxine-Pyrimethamine on Antibody Responses to Erythrocytic-Stage Antigens in Infants in Mozambique. Clin Vaccine Immunol.

[14] Coldiron ME, Von Seidlein L, Grais RF (2017). Seasonal malaria chemoprevention: successes and missed opportunities. Malar J.

[15] Pitt C, Diawara H, Ouédraogo Dimlawendé J, Diarra S, Kaboré Habibou, Kouéla Kibsbila, Traoré Abdoulaye, Dicko A, Konaté Amadou T, Chandramohan D, Diallo DA, Greenwood B, Conteh L (2012). Intermittent preventive treatment of malaria in children: a qualitative study of community perceptions and recommendations in Burkina Faso and Mali. PLoS One.

[16] Ding J, Coldiron Matthew E, Assao Bachir, Guindo Ousmane, Blessborn Daniel, Winterberg Markus, Grais Rebecca F, Koscalova Alena, Langendorf Celine, Tarning Joel (2020). Adherence and Population Pharmacokinetic Properties of Amodiaquine When Used for Seasonal Malaria Chemoprevention in African Children. Clin Pharmacol Ther.

[17] van Lenthe Marit, van der Meulen Renske, Lassovski Maryvonne, Ouabo Adelaide, Bakula Edwige, Badio Colette, Cibenda Deogratias, Okell Lucy, Piriou Erwan, Grignard Lynn, Lanke Kjerstin, Rao Bhargavi, Bousema Teun, Roper Cally (2019). Markers of sulfadoxine-pyrimethamine resistance in Eastern Democratic Republic of Congo; implications for malaria chemoprevention. Malar J.

[18] Allen EN, Little F, Camba T, Cassam Y, Raman J, Boulle A, Barnes KI (2009). Efficacy of sulphadoxine-pyrimethamine with or without artesunate for the treatment of uncomplicated Plasmodium falciparum malaria in southern Mozambique: a randomized controlled trial. Malar J.

[19] Gupta H, Macete E, Bulo H, Salvador C, Warsame M, Carvalho E, Ménard Didier, Ringwald P, Bassat Q, Enosse S, Mayor A (2018). Drug-Resistant Polymorphisms and Copy Numbers in Plasmodium falciparum, Mozambique, 2015. Emerg Infect Dis.

[20] Grais R, Laminou Ibrahim M, Woi-Messe Lynda, Makarimi Rockyath, Bouriema Seidou H, Langendorf Celine, Amambua-Ngwa Alfred, D'Alessandro Umberto, Guérin Philippe J, Fandeur Thierry, Sibley Carol H (2018). Molecular markers of resistance to amodiaquine plus sulfadoxine-pyrimethamine in an area with seasonal malaria chemoprevention in south central Niger. Malar J.

[21] Chico RM, Cano J, Ariti C, Collier TJ, Chandramohan D, Roper C, Greenwood B (2015). Influence of malaria transmission intensity and the 581G mutation on the efficacy of intermittent preventive treatment in pregnancy: systematic review and meta-analysis. Trop Med Int Health.

[22] Gutman J, Kalilani Linda, Taylor Steve, Zhou Zhiyong, Wiegand Ryan E, Thwai Kyaw L, Mwandama Dyson, Khairallah Carole, Madanitsa Mwayi, Chaluluka Ebbie, Dzinjalamala Fraction, Ali Doreen, Mathanga Don P, Skarbinski Jacek, Shi Ya Ping, Meshnick Steve, ter Kuile Feiko O (2015). The A581G Mutation in the Gene Encoding Plasmodium falciparum Dihydropteroate Synthetase Reduces the Effectiveness of Sulfadoxine-Pyrimethamine Preventive Therapy in Malawian Pregnant Women. J Infect Dis.

[23] Harrington W, Mutabingwa Theonest K, Kabyemela Edward, Fried Michal, Duffy Patrick E (2011). Intermittent treatment to prevent pregnancy malaria does not confer benefit in an area of widespread drug resistance. Clin Infect Dis.

[24] Desai M, Gutman Julie, Taylor Steve M, Wiegand Ryan E, Khairallah Carole, Kayentao Kassoum, Ouma Peter, Coulibaly Sheick O, Kalilani Linda, Mace Kimberly E, Arinaitwe Emmanuel, Mathanga Don P, Doumbo Ogobara, Otieno Kephas, Edgar Dabira, Chaluluka Ebbie, Kamuliwo Mulakwa, Ades Veronica, Skarbinski Jacek, Shi Ya Ping, Magnussen Pascal, Meshnick Steve, Ter Kuile Feiko O (2016). Impact of Sulfadoxine-Pyrimethamine Resistance on Effectiveness of Intermittent Preventive Therapy for Malaria in Pregnancy at Clearing Infections and Preventing Low Birth Weight. Clin Infect Dis.

[25] Okell L, Griffin Jamie T, Roper Cally (2017). Mapping sulphadoxine-pyrimethamine-resistant Plasmodium falciparum malaria in infected humans and in parasite populations in Africa. Sci Rep.

[26] National Malaria Control Programme Ministry of Health Mozambique (2019). Procedure Manual. Routine analysis of National Malaria Control Programme data quality.

[27] World Health Organization (2012). Seasonal malaria chemoprevention (SMC) for plasmodium falciparum malaria control in highly seasonal transmission areas of the Sahel sub-region of Africa. WHO Policy Recommendation.

[28] Antwi GD, Bates LA, King R, Mahama PR, Tagbor H, Cairns M, Newell JN (2016). Facilitators and Barriers to Uptake of an Extended Seasonal Malaria Chemoprevention Programme in Ghana: A Qualitative Study of Caregivers and Community Health Workers. PLoS One.

[29] Curran GM, Bauer M, Mittman B, Pyne JM, Stetler C (2012). Effectiveness-implementation hybrid designs: combining elements of clinical effectiveness and implementation research to enhance public health impact. Med Care.

[30] Wolfenden L, Williams CM, Wiggers J, Nathan N, Yoong SL (2016). Improving the translation of health promotion interventions using effectiveness-implementation hybrid designs in program evaluations. Health Promot J Austr.

[31] Lauria M, Fiori Kevin P, Jones Heidi E, Gbeleou Sesso, Kenkou Komlan, Agoro Sibabe, Agbèrè Abdourahmane Diparidé, Lue Kelly D, Hirschhorn Lisa R (2019). Assessing the Integrated Community-Based Health Systems Strengthening initiative in northern Togo: a pragmatic effectiveness-implementation study protocol. Implement Sci.

[32] World Health Organization (2017). World Malaria Program. Minutes of the Technical Expert Group on Drug Efficacy and Response.

[33] Centro de Investigação em Saúde de Manhiça (2020). Unpublished data. Unpublished data.

[34] Ross A, Smith Thomas (2010). Interpreting malaria age-prevalence and incidence curves: a simulation study of the effects of different types of heterogeneity. Malar J.

[35] Venkataramani Atheendar S, Bor Jacob, Jena Anupam B (2016). Regression discontinuity designs in healthcare research. BMJ.

[36] Marshall M (1996). Sampling for qualitative research. Fam Pract.

[37] Green J, Thorogood N (2009). Qualitative Methods for Health Research.

[38] O’Reilly M, Parker N (2012). ‘Unsatisfactory Saturation’: a critical exploration of the notion of saturated sample sizes in qualitative research. Qualitative Research.

[39] Rapley TJ (2016). The art(fulness) of open-ended interviewing: some considerations on analysing interviews. Qualitative Research.

[40] Arskey H, Knight P (1999). Interviewing for social scientists.

[41] Brocki J, Wearden Aj (2007). A critical evaluation of the use of interpretative phenomenological analysis (IPA) in health psychology. Psychology & Health.

[42] Corbin J, Strauss A (2008). Basics of qualitative research: Techniques and procedures for developing grounded theory.

[43] Compaoré Rachidatou, Yameogo Maurice Wambi Evariste, Millogo Tieba, Tougri Halima, Kouanda Seni (2017). Evaluation of the implementation fidelity of the seasonal malaria chemoprevention intervention in Kaya health district, Burkina Faso. PLoS One.

[44] Konaté AT, Yaro JB, Ouédraogo AZ, Diarra A, Gansané A, Soulama I, Kangoyé DT, Kaboré Y, Ouédraogo E, Ouédraogo A, Tiono AB, Ouédraogo IN, Chandramohan D, Cousens S, Milligan PJ, Sirima SB, Greenwood B, Diallo DA (2011). Intermittent preventive treatment of malaria provides substantial protection against malaria in children already protected by an insecticide-treated bednet in Burkina Faso: a randomised, double-blind, placebo-controlled trial. PLoS Med.

[45] Dicko A, Diallo AI, Tembine I, Dicko Y, Dara N, Sidibe Y, Santara G, Diawara H, Conaré T, Djimde A, Chandramohan D, Cousens S, Milligan PJ, Diallo DA, Doumbo OK, Greenwood B (2011). Intermittent Preventive Treatment of Malaria Provides Substantial Protection against Malaria in Children Already Protected by an Insecticide-Treated Bednet in Mali: A Randomised, Double-Blind, Placebo-Controlled Trial. PLoS Med.

[46] Diawara F, Steinhardt Laura C, Mahamar Almahamoudou, Traore Tiangoua, Kone Daouda T, Diawara Halimatou, Kamate Beh, Kone Diakalia, Diallo Mouctar, Sadou Aboubacar, Mihigo Jules, Sagara Issaka, Djimde Abdoulaye A, Eckert Erin, Dicko Alassane (2017). Measuring the impact of seasonal malaria chemoprevention as part of routine malaria control in Kita, Mali. Malar J.

[47] Druetz T, Corneau-Tremblay Nicolas, Millogo Tieba, Kouanda Seni, Ly Antarou, Bicaba Abel, Haddad Slim (2018). Impact Evaluation of Seasonal Malaria Chemoprevention under Routine Program Implementation: A Quasi-Experimental Study in Burkina Faso. Am J Trop Med Hyg.

[48] Nonvignon J, Aryeetey Genevieve Cecilia, Issah Shamwill, Ansah Patrick, Malm Keziah L, Ofosu Winfred, Tagoe Titus, Agyemang Samuel Agyei, Aikins Moses (2016). Cost-effectiveness of seasonal malaria chemoprevention in upper west region of Ghana. Malar J.

